# Benchmarks in Liver Resection for Intrahepatic Cholangiocarcinoma

**DOI:** 10.1245/s10434-023-14880-8

**Published:** 2024-01-12

**Authors:** Laura Alaimo, Yutaka Endo, Giovanni Catalano, Andrea Ruzzenente, Luca Aldrighetti, Matthew Weiss, Todd W. Bauer, Sorin Alexandrescu, George A. Poultsides, Shishir K. Maithel, Hugo P. Marques, Guillaume Martel, Carlo Pulitano, Feng Shen, François Cauchy, Bas Groot Koerkamp, Itaru Endo, Minoru Kitago, Timothy M. Pawlik

**Affiliations:** 1 Department of Surgery, The Ohio State University Wexner Medical Center and James Comprehensive Cancer Center, Columbus, OH USA; 2 Department of Surgery, University of Verona, Verona, Italy; 3 Department of Surgery, Ospedale San Raffaele, Milan, Italy; 4 Department of Surgery, Johns Hopkins Hospital, Baltimore, MD USA; 5 Department of Surgery, University of Virginia, Charlottesville, VA USA; 6 Department of Surgery, Fundeni Clinical Institute, Bucharest, Romania; 7 Department of Surgery, Stanford University, Stanford, CA USA; 8 Department of Surgery, Emory University, Atlanta, GA USA; 9 Department of Surgery, Curry Cabral Hospital, Lisbon, Portugal; 10 Department of Surgery, University of Ottawa, Ottawa, ON Canada; 11 Department of Surgery, Royal Prince Alfred Hospital, University of Sydney, Sydney, NSW Australia; 12 Department of Surgery, Eastern Hepatobiliary Surgery Hospital, Shanghai, China; 13 Department of Hepatobiliopancreatic Surgery and Liver Transplantation, AP-HP, Beaujon Hospital, Clichy, France; 14 Department of Surgery, Erasmus University Medical Centre, Rotterdam, The Netherlands; 15 Department of Gastroenterological Surgery, Yokohama City University School of Medicine, Yokohama, Japan; 16 Department of Surgery, Keio University, Tokyo, Japan

## Abstract

**Introduction:**

Benchmarking in surgery has been proposed as a means to compare results across institutions to establish best practices. We sought to define benchmark values for hepatectomy for intrahepatic cholangiocarcinoma (ICC) across an international population.

**Methods:**

Patients who underwent liver resection for ICC between 1990 and 2020 were identified from an international database, including 14 Eastern and Western institutions. Patients operated on at high-volume centers who had no preoperative jaundice, ASA class <3, body mass index <35 km/m^2^, without need for bile duct or vascular resection were chosen as the benchmark group.

**Results:**

Among 1193 patients who underwent curative-intent hepatectomy for ICC, 600 (50.3%) were included in the benchmark group. Among benchmark patients, median age was 58.0 years (interquartile range [IQR] 49.0–67.0), only 28 (4.7%) patients received neoadjuvant therapy, and most patients had a minor resection (*n* = 499, 83.2%). Benchmark values included ≥3 lymph nodes retrieved when lymphadenectomy was performed, blood loss ≤600 mL, perioperative blood transfusion rate ≤42.9%, and operative time ≤339 min. The postoperative benchmark values included TOO achievement ≥59.3%, positive resection margin ≤27.5%, 30-day readmission ≤3.6%, Clavien-Dindo III or more complications ≤14.3%, and 90-day mortality ≤4.8%, as well as hospital stay ≤14 days.

**Conclusions:**

Benchmark cutoffs targeting short-term perioperative outcomes can help to facilitate comparisons across hospitals performing liver resection for ICC, assess inter-institutional variation, and identify the highest-performing centers to improve surgical and oncologic outcomes.

**Supplementary Information:**

The online version contains supplementary material available at 10.1245/s10434-023-14880-8.

Cholangiocarcinoma is the second most common primary liver cancer after hepatocellular carcinoma (HCC), with intrahepatic cholangiocarcinoma (ICC) accounting for 10% of all biliary tract cancers (BTC) and 15% of all primary liver tumors.^1,2^ The incidence of ICC is increasing globally with as many as two cases per 100,000 people per year in Western countries.^3^ Unfortunately, due to an often late diagnosis and aggressive tumor behavior, a large proportion of patients are not eligible for curative-intent surgery.^4^ Among patients with inoperable disease, the prognosis is particularly poor with a reported median overall survival (OS) between 3 and 12 months.^5^ In contrast, liver resection with lymphadenectomy is the cornerstone of curative-intent treatment for patients with resectable ICC. Resection may involve a major hepatectomy with or without associated vascular or extrahepatic bile duct resection to achieve negative microscopic margins (i.e., R0 resection). In turn, these complex resections may be associated with a high risk of morbidity and mortality even at high-volume centers.^6,7^ Furthermore, the surgical approach to ICC has changed over the past decade. For example, lymphadenectomy has become a standard procedure, and the minimally invasive approach was only recently introduced.^1,2^ These recent innovations led to variations among different institution practices due to corresponding differences in surgeon expertise and technique. The surgical management of ICC is a technically demanding procedure that necessitates considerable expertise to conform to high-quality operative standards. For this reason, it is important to establish specific ICC surgical quality outcomes to assess low- versus high-quality outcomes among low- versus high-volume centers.

Currently, there are no established reference values to assess the quality of outcomes related to liver surgery for ICC. The lack of data to compare centers can lead to unproven claims of superiority and make conclusive comparisons among centers impossible. Numerous surgical outcomes have been used to indicate institutional “quality” for the treatment of various diseases.^8–10^ Individual metrics do not fully represent, however, the overall quality of surgical care despite their importance in isolated domains of perioperative care.^11^ In addition, among cancer patients who often require complex multidisciplinary care, the need to comparatively assess quality at different centers may take on even greater importance. Benchmarking is a methodology employed to establish comprehensive quality measures and has been applied to compare clinical outcomes against key performance indicators to evaluate the best possible performance among “benchmark cases” representing the “best case scenario.”^12–15^ The benchmark value represents the best possible outcome, while the gap between the benchmark and actual performance signifies the potential for improvement.^14,16^ As such, the goal of surgical benchmarking is to find hospitals that can serve as a standard reference to assess other center outcomes related to patient care. To this point, benchmarking can drive more efficient structures and workflows to improve patient outcomes.^10,14,16–18^

Defining benchmark values as a reference for liver resection of ICC may serve as a means for institutions to assess their surgical performance, improve outcomes, and help move toward performing higher-quality surgery. Therefore, the goal of the current international multi-institutional study was to identify clinically relevant perioperative outcomes related to curative-intent liver resection for ICC and establish benchmark values that can be applied to a heterogeneous population worldwide.

## Methods

### Study Population

Patients who underwent curative-intent liver resection for intrahepatic cholangiocarcinoma (ICC) between 1990 and 2020 were identified from an international multi-institutional database that included 14 institutions from Eastern and Western countries (Table 1). Patients who received palliative treatment were excluded. The Institutional Review Board of each participating center and The Ohio State University approved the study.

**Table 1:** Participating institutions

Institution	
*Eastern*	
Eastern Hepatobiliary Surgery Hospital	Shanghai, China
Yokohama City University	Yokohama, Japan
Keio University	Tokyo, Japan
*Western*	
*USA/Canada*	
John Hopkins University	Baltimore, MA, USA
Stanford University	Stanford, CA, USA
Emory University	Atlanta, GA, USA
University of Virginia	Charlottesville, USA
University of Ottawa	Ottawa, Canada
*Europe*	
University of Verona	Verona, Italy
Ospedale San Raffaele	Milan, Italy
Beaujon Hospital	Clichy, France
Fundeni Clinical Institute	Bucharest, Romania
Curry Cabral Hospital	Lisbon, Portugal
Erasmus Medical Center	Rotterdam, Netherlands

Data on patient demographics (i.e., age, sex, body mass index [BMI], ASA physical status classification, the presence of cirrhosis, preoperative jaundice, albumin-bilirubin [ALBI] score, and carbohydrate antigen 19-9 [CA19-9]), tumor-related factors (i.e., size of the largest lesion [cm], number of lesions, tumor burden score [TBS], grade of differentiation, lymphovascular invasion, and perineural invasion), and treatment data (i.e., neoadjuvant therapy, extent of resection, extended resection, vascular resection, and bile duct resection) were collected. The type of hepatectomy was defined according to the “New World” terminology for hepatectomy.^19^ The extent of resection was classified as minor (<3 Couinaud segments) or major (≥3 Couinaud segments).^20^ TBS, a concise metric of ICC tumor burden, was calculated based on the formula: [TBS^2^ = (maximum tumor diameter)^2^ + (number of tumors)^2^].^21^

### Definitions

The benchmark group consisted of patients who underwent surgery at high-volume centers and met the following criteria: absence of preoperative jaundice, ASA class <3, body mass index <35 km/m^2^, and no requirement for bile duct resection, or vascular resection.^15^ The benchmark values were established for various outcome measures, including the number of retrieved lymph nodes (in the setting of lymphadenectomy), estimated intraoperative blood loss, perioperative blood transfusion, operative time, textbook oncologic outcome (TOO), and its constituent components, as well as the length of hospital stay (LOS). TOO was defined as achieving negative resection margins (R0 resection), with no occurrences of 30-day readmission, severe complications, or 90-day mortality. The severity of postoperative complications was graded according to the Clavien-Dindo classification, and Clavien-Dindo ≥3 complications were defined as severe complications.^22^ Each benchmark value was calculated individually for each center, and the benchmark range was determined as the span from the 25th to the 75th percentile based on the median values across centers. Benchmark cutoff points were derived from either the 75th percentile for values indicating worse outcomes (i.e., estimated intraoperative blood loss, perioperative blood transfusion, operative time, positive resection margin, severe complications, 90-day mortality) or the 25th percentile for indicators of favorable outcomes (i.e., number of lymph nodes retrieved, and TOO) based on median values from each participating center.^23^

### Statistical Analysis

For descriptive statistics, categorical variables were reported as frequencies (%) and compared by using the χ^2^ test or Fisher exact test, as appropriate. Continuous variables were summarized as median (interquartile range [IQR]) and compared using the Mann-Whitney *U* test. Multiple imputations with chain equations (MICE) were utilized to address missingness.^24^ Survival probabilities were estimated by utilizing Kaplan-Meier curves and compared by using the log-rank test. Pearson correlation was performed to compute correlation coefficients between measured variables. All statistical tests were two-sided, and the significance level was set at α = 0.05. Statistical analyses were conducted in R version 4.2.0 (R Project for Statistical Computing) and SPSS version 28.0 (IBM Corporation).

## Results

### Patient Characteristics

Among the 14 participating centers (USA/Canada: n = 5, Europe: n = 6, Asia: n = 3), 1,193 patients underwent liver resection for ICC (Supplementary Table 1). Median patient age was 61.0 years (IQR 52.9-69.6), and 654 (54.8%) individuals were male. Notably, 30% of patients (n = 358) were categorized as ASA ≥3, whereas only 4% had a BMI ≥35 kg/m^2^ (n = 48). A subset of 107 (9.0%) patients received neoadjuvant treatment; 134 (11.2%) patients underwent extended hepatectomy, 244 (20.5%) had concurrent vascular resection, and 160 (13.4%) patients underwent bile duct resection. Median duration of the surgical procedure was 233.0 minutes (IQR 138.0–361.0). Estimated blood loss was 450.0 mL (IQR 200.0–800.0), and 602 (50.5%) patients had a blood transfusion. On pathology, a majority of patients had a single lesion (n = 993, 83.2%) with a median tumor size of 6.0 cm (IQR 4.0–8.6); median TBS was 6.1 (IQR 4.1–8.8). Approximately one-half of patients underwent lymphadenectomy (n = 626, 52.5%) at the time of hepatic resection; among these individuals, 22.9% had nodal metastases (n = 273, 22.9%). TOO was achieved in 67.1% of patients (n = 800), and median length of hospital stay was 13.0 days (IQR 8.0–18.0).

### Postoperative Outcomes of Patients in the Benchmark Group

**Fig. 1:**
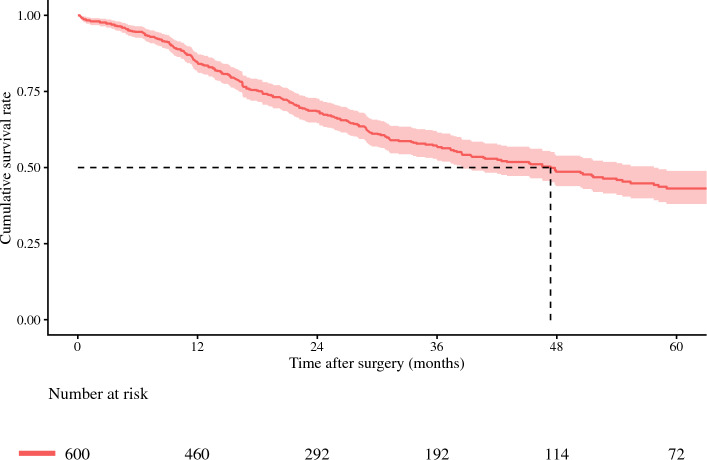
Overall survival in the benchmark group

A total of 600 (50.3%) patients met the criteria and were categorized as benchmark cases. Among individuals in the benchmark cohort, 52 (8.7%) patients experienced a Clavien–Dindo grade ≥ IIIa complication (Table 2); ten (1.7%) patients died within 90 days of surgery. Median length of postoperative hospital stay was 13 days (IQR 9–17). Median operation time and blood loss were 180.0 minutes (IQR 108.0–298.0) and 300.0 ml (IQR 200.0–600.0), respectively. A perioperative blood transfusion was administered to 259 (43.2%) patients. With a median follow-up of 19.0 months (IQR 9.1–27.6), median OS in the benchmark cohort was 47.4 months (95% CI 38.5–57.7 months); 1-, 3-, and 5-year OS rates were 84.5%, 57.0%, and 43.1%, respectively (Fig. 1).

**Table 2:** Comparison between benchmark and nonbenchmark cohort

	Benchmark	Nonbenchmark	*p*
	*n* = 600	*n* = 593	
*Patient demographics*			
Age, years, median (IQR)	58.0 (49.0, 67.0)	63.1 (55.2, 71.0)	<0.001
Sex, male, n (%)	347 (57.8)	307 (51.8)	0.04
Year of surgery, 2011–2020, n (%)	321 (53.5)	288 (48.6)	0.10
Cirrhosis, n (%)	84 (14.0)	53 (8.9)	<0.01
Body mass index, kg/m^2^, median (IQR)	24.5 (22.0, 27.2)	25.3 (22.7, 28.8)	<0.001
ALBI score, median (IQR)	−2.73 (−3.00, −2.44)	−2.40 (−2.74, −1.96)	<0.001
CA19-9, UI/mL, median (IQR)	44.0 (15.9, 182.9)	65.0 (22.0, 400.0)	<0.001
*Tumor characteristics*			
Largest tumor size, cm, median (IQR)	6.0 (4.0, 8.0)	6.0 (4.0, 9.0)	0.22
No. lesions, median (IQR)			
Single	519 (86.5)	474 (79.9)	<0.01
Multiple	81 (13.5)	119 (20.1)	
Tumor burden score, median (IQR)	6.1 (4.1, 8.3)	6.2 (4.1, 9.1)	0.11
Morphology, periductal infiltrating type, n (%)	25 (4.2)	143 (24.1)	<0.001
Histological grade, poor differentiated, n (%)	80 (13.3)	140 (23.6)	<0.001
Lymphovascular invasion, n (%)	176 (29.3)	238 (40.1)	<0.001
Perineural invasion, n (%)	87 (14.5)	189 (31.9)	<0.001
*Treatment data*			
Neoadjuvant therapy, n (%)	28 (4.7)	79 (13.3)	<0.001
Major resection, n (%)	101 (16.8)	209 (35.2)	<0.001
*Outcomes*			
Operation time, min, median (IQR)	180.0 (108.0, 298.0)	300.0 (200.0, 459.0)	<0.001
Estimated blood loss, ml, median (IQR)	300.0 (200.0, 600.0)	600.0 (300.0, 1100.0)	<0.001
Blood transfusion, n (%)	259 (43.2)	343 (57.8)	<0.001
Lymphadenectomy, n (%)	257 (42.8)	369 (62.2)	<0.001
Lymph node metastases, n (%)	92 (15.3)	181 (30.5)	<0.001
Textbook Oncological Outcome, n (%)	478 (79.7)	322 (54.3)	<0.001
Positive margin resection, n (%)	77 (12.8)	133 (22.4)	<0.001
30-day readmission, n (%)	8 (1.3)	83 (14.0)	<0.001
Severe complication, n (%)	52 (8.7)	115 (19.4)	<0.001
90-day mortality, n (%)	10 (1.7)	28 (4.7)	<0.01
Length of stay, days, median (IQR)	13 (9, 17)	12 (7, 20)	0.04

*ALBI score* albumin-bilirubin score; *IQR* interqurtile range

### Comparison Between Benchmark and Nonbenchmark Groups

There were marked differences among patients included in the benchmark versus nonbenchmark cohorts. For example, benchmark patients typically had more favorable clinicopathologic characteristics related to demographic factors, such as age, ALBI score, as well as tumor characteristics, such as CA19-9 levels, periductal infiltrating morphology, poor differentiation, lymphovascular, and perineural invasion, as well as lymph node metastases (Table 1). As a result, only a few benchmark patients received neoadjuvant chemotherapy and underwent major resection. Notably, patients in the nonbenchmark cohort had a higher incidence of major complications, positive resection margin, 30-day readmission, 90-day mortality, and ultimately lower rates of TOO achievement versus patients in the benchmark cohort. Patients categorized into the benchmark group had improved overall survival compared with individuals in the nonbenchmark cohort (5-year OS: benchmark, 43.1% vs. nonbenchmark, 35.5%, *p* < 0.001) (Supplementary Fig. 1).

### Benchmark Values

Overall benchmark values are reported in Table 3. The intraoperative benchmark values were ≥3 lymph nodes retrieved during lymphadenectomy, EBL ≤600 mL, perioperative blood transfusion rate ≤42.9%, and operative time ≤339 min. Postoperative benchmark values were a rate TOO achievement of ≥59.3%, positive resection margins ≤27.5%, Clavien-Dindo III or more complications ≤14.3%, 30-day readmission ≤3.6%, and 90-day mortality ≤4.8%. In addition, specific benchmarks were defined relative to the extent of liver resection (Supplementary Table 2). The number of lymph nodes retrieved did not differ between patients who underwent minor versus major hepatectomy. Of note, EBL, perioperative blood transfusion, operative time, TOO achievement, likelihood of positive margin resection, and hospital stay were higher in the major hepatectomy cohort. In contrast, Clavien-Dindo III or higher complications, 30-day readmission, and 90-day mortality were higher in the minor hepatectomy cohort.

**Table 3:** Benchmark values in liver resection for intrahepatic cholangiocarcinoma

Parameter	25th percentile	50th percentile	75th percentile	Benchmark values
No. lymph nodes retrieved	3.0	4.0	6.0	3.0
Estimated intraoperative blood loss, ml	250.0	450.0	600.0	600.0
Perioperative blood transfusion, %	22.9	33.3	42.9	42.9
Operative time, min	180.0	240.0	339.0	339.0
Textbook oncological outcome, %	59.3	66.7	83.3	59.3
Positive margin resection, %	7.7	19.6	27.5	27.5
30-day readmission, %	0	0	3.6	3.6
Severe complication, %	3.7	7.1	14.3	14.3
90-day mortality, %	0	0	4.8	4.8
Postoperative hospital stay, median, days	7.0	9.0	14.0	14.0

### Institutional Geographical Variations, Practice Patterns, and Outcomes

**Fig. 2:**
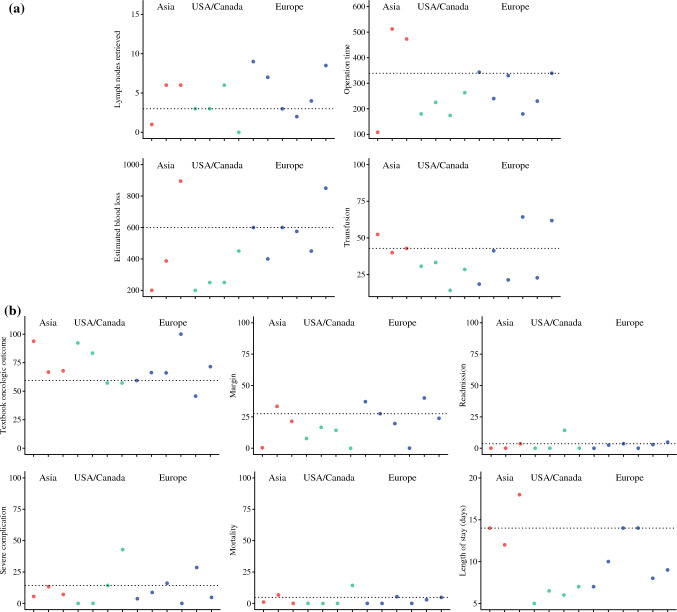
Comparison between institutions’ rates of intraoperative findings (**a**), such as the number of lymph nodes retrieved, operation time, blood loss, and transfusion rate, and postoperative outcomes (**b**), such as textbook oncologic outcome, positive resection margin rate, 30-day readmission rate, severe complications rate, 90-day mortality rate, and length of stay

Figure 2 depicts substantial variations in benchmark values. For instance, the number of retrieved lymph nodes, operation time, estimated blood loss, and the utilization of transfusion demonstrated wide variation among institutions (number of retrieved nodes: 0–9; operation time: 108.0–521.0 minutes; EBL: 200–895 ml; and transfusion rate: 14.3–64.3%). Also, the incidence of margin-positive resection and severe complications varied considerably between institutions. Specifically, margin-positive resection ranged from 0% to 40.0%, and the incidence of severe complications varied from 0% to 42.9%. In turn, these variations drove major differences in TOO achievement rate at different centers (45.7–100%). Of note, there was considerable variation in the length of stay ranging from 5.0 to 18.0 days.

### Comparison Among Benchmark Patients with Negative Versus Positive Resection Margin

**Fig. 3:**
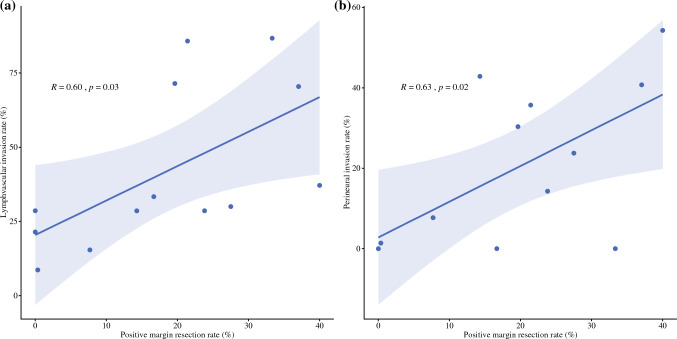
Correlation between institutional R1 resection rate and lymphvascular invasion (**a**) and perineural invasion (**b**)

To further examine possible factors contributing to variations in achievement of a negative resection margin, a subset analysis was conducted among benchmark cases that were stratified based on margin status. Notably, patients with a positive resection margin were more likely to be older (65.0 years vs. 57.0 years) and female (61.0% vs. 39.4%), as well as have a higher TBS (7.1 vs. 6.1), poor histological grade (23.4% vs. 11.9%), lymphovascular (59.7% vs. 24.9%), and perineural (37.7% vs. 11.1%) invasion (all *p* < 0.05) (Supplementary Table 3). Hospital-level analysis examining the correlation between an R1 resection and the presence of lymphovascular and perineural invasion demonstrated a positive correlation (lymphovascular invasion: *R* = 0.60, *p* = 0.03; perineural invasion: *R* = 0.63, *p* = 0.02; Fig. 3).

## Discussion

Given a rising global incidence with a particularly dramatic increase in North America and Europe, there has been increased interest in the clinical and surgical management of ICC.^3^ While curative-intent surgery is the standard treatment option for resectable ICC, liver resection can be associated with high morbidity and mortality even at high-volume centers.^4,5^ Quality improvement, especially for high-risk, high-morbidity procedures, requires measuring and tracking patient outcomes, as well as review of these data to ensure certain “standards” are being met. Often, local outcomes from a given center are compared with regional or national data to ensure a minimal standard relative to outcomes at other hospitals. This process can, however, lead to unfair comparisons of heterogeneous populations across different institutions with different referral patterns and practices.^25^ Benchmarking is a relatively novel tool used to identify best practices and benchmarks in quality domains for institutional-level improvement initiatives. In 2022, a conference was held in Zurich to discuss the effectiveness of benchmarking in relation to surgical outcomes.^12^ The conference emphasized the need to integrate processes for public data reporting and research on benchmarking surgical outcomes.^12,25^ Although several studies have attempted to establish benchmarks for various surgical hepatobiliary procedures, to the best of our knowledge, few reports have specifically focused on the surgical treatment of ICC. In this regard, the current study was important, because the benchmark methodology was applied to a multi-institutional cohort of patients with ICC treated at some of the most experienced hepatobiliary centers worldwide. Intraoperative benchmark values included ≥3 lymph nodes retrieved, blood loss ≤600 mL, perioperative blood transfusion utilization ≤42.9%, and operative time ≤339 min. In addition, postoperative benchmark standards were identified, such as achievement of TOO ≥59.3%, positive margin resection ≤27.5%, Clavien-Dindo III or more complications ≤14.3%, hospital stay ≤14 days, 30-day readmission ≤3.6%, and 90-day mortality ≤4.8%. In turn, benchmarking data derived from this large, international database can be used to establish surgical standards relative to resection of ICC.

Accurate measurement of quality, as well as identifying specific targets to enhance operational efficiency, are important to streamline the surgical workflow and drive process improvement. Comparison of quality among local hospitals with national-based “average” outcomes using electronic health records and national administrative databases has inherent limitations and may not be adequate to identify opportunities for quality improvement. Rather, benchmarking involves a continual process of self-evaluation and comparison with other best-practice institutions.^26^ Previous reports on benchmarking for liver resection have been limited by assessing specific operative approaches or procedures and included a wide variety of diagnoses.^13,17,27–30^ In the current study, we specifically analyzed only patients who underwent curative-intent surgery for ICC at specialized centers. Of note, the benchmark value among these high-volume liver centers was 14.9% for severe complications. Perhaps not surprisingly, this benchmark was higher than the value reported for hepatectomies performed on healthy living donors.^13^ In contrast, compared with benchmark cohorts of patients who underwent ALPSS or liver transplantation, the benchmarks reported in the current study were lower for morbidity and severe complications.^30,31^ These data highlight the need for a risk-adjusted comparison of outcomes based on surgical indication to compare results associated with major hepatectomy. In turn, hospital quality programs can drive improvement in safety and outcomes by targetting these surgical benchmark values. Quality initiatives may involve improvement in quality and surgical delivery at same centers of care, as well as increased efforts to regionalize complex liver surgery to meet benchmarks.^10^ Use of benchmarking may improve quality of surgical outcomes, as well as reduced costs associated with a surgical episode of care.^26,32^

One interesting finding of the current study was the marked differences in intraoperative metrics even among high-volume hepatobiliary institutions. For instance, the number of retrieved lymph nodes, operation time, estimated blood loss, and the use of blood transfusions varied widely among institutions. The underlying reasons for these variations are undoubtedly multifaceted but may be related to differences in patient selection, surgical techniques, such as liver parenchymal dissection methods, use of the Pringle maneuver, as well as different proportions of cases that were performed using an open versus a minimally invasive (e.g., laparoscopic, robotic) approach.^33–35^ Furthermore, while the AJCC 8^th^ edition has established six lymph nodes as the optimal number to be evaluated, the routine use of lymphadenectomy, as well as the number of lymph nodes harvested remains debated.^36^ The controversity around lymphadenectomy further highlights the need for benchmarking in the surgical management of ICC.^37,38^ Another perioperative metric that varied considerably was the length of postoperative hospital stay (Supplementary Fig. 2b). Previous studies had similarly noted that benchmarking length of stay can be challenging and confounded by geography. For example, Mueller et al. noted that institutions in Asian countries had markedly longer LOS versus non-Asian countries.^15^ Variations in global healthcare and insurance systems—as well as cultural differences—likely influence the average duration of postoperative hospital stays.^39^ Consequently, while benchmarking may be useful to compare length-of-stay with certain geographic locations, this metric is not likely useful to compare centers in different countries.^12^

Advances in surgical technique and patient optimization have improved perioperative and oncological outcomes for patients undergoing liver resection. Despite these advances, liver resection for ICC can be characterized by a high incidence of R1-resection with the final pathology demonstrating microscopic tumor invasion within the resection margin that was not apparent during surgery; R1 margin status can be particularly high among patients with tumors that are large or centrally located.^37,40^ Achieving an R0 resection is important, however, to facilitate the best oncological outcomes for patients.^41^ In the current study, the incidence of a positive resection margin status at the different centers varied from 0% to 42.9%. These data serve to emphasize the need for a higher achievement of a negative surgical margin compared with the benchmark value. Of note, positive margin status correlated with lymphovascular and perineural invasion (Fig. 3). In addition, in clinical practice, some patients are not candidates for more extensive resection to achieve an R0 margin due to the impairment of liver function or physical status. As such, variations in R0 margin status at the different centers may have been related to differences in underlying tumor biology, as well as the characteristics of the target patient population. In turn, the definition of "benchmark cases" may need to include other factors to represent the ideal or best-case scenario patient population. Taking other pathologic—or even genetic—factors into account may be needed in the future to define benchmark cases to reflect more accurately the complexity of this patient population.

The current paper should be considered in light of several limitations. Because of the retrospective design, selection and reporting biases may have influenced the results. The inclusion of patients from multiple centers across the globe was a strength, allowing for the establishment of global benchmark values. There was, however, the possibility of facility and regional variation in patient selection for surgery and perioperative patient optimization. The outcomes of liver resections also may have been influenced by changes in clinical practices over time and between centers. Patients who were considered incurable in the past can now undergo aggressive surgical resection, and more effective chemotherapy is available in high-volume centers. These treatment options may result in less favorable short-term outcomes. In addition, having a diverse patient population undergoing liver resection for ICC may have caused some biases due to varying surgical complexity and patient health status, which can impact short-term results. Specifically, patients may have different health status, comorbidities, malignancies, and a history of preoperative treatment, including neoadjuvant chemotherapy. However, given that we assessed “all comer” ICC patients, the current benchmark values likely reflect the complex multimodal care delivered at tertiary referral centers. Although certain geographic areas were unrepresented in the collaborative, the current study did represent one of the largest, international, multi-institutional ICC worldwide cohorts. In turn, the results should be interpreted with this limitaiton in mind, and future studies should seek to include centers in other geographic (i.e., Africa, South America) to validate the global benchmarks reported in the current study.

## Conclusions

Data from this large, multi-institutional study provides reference benchmark values for major hepatic surgery among patients undergoing liver resection of ICC. These benchmarks may facilitate comparison of outcomes among different patient cohorts, aiding in the assessment of surgical performance and oncological efficacy in surgical management of ICC across the globe.

### Supplementary Information

Below is the link to the electronic supplementary material.

Supplementary file1 (DOCX 28 KB)
