# Supplementary material for: Benchmarks in Liver Resection for Intrahepatic Cholangiocarcinoma
Source: Ann Surg Oncol. 2024 Jan 12;31(5):3043–52. doi: 10.1245/s10434-023-14880-8 (PMC10997542; doi:10.1245/s10434-023-14880-8)
Supplement: Supplementary file 1 — Supplementary file1 (DOCX 28 KB) [file 10434_2023_14880_MOESM1_ESM.docx]

**Supplementary Table 1** Background of all analytic cohort

|  | All patients |
| --- | --- |
|  | n=1,193 |
| ***Patient demographics*** |  |
| Age, y, median (IQR) | 61.0 (52.9-69.6) |
| Sex, male, n (%) | 654 (54.8) |
| ASA-PS, ≥3, n (%) | 358 (30.0) |
| Year of surgery, 2011-2020, n (%) | 609 (51.0) |
| Cirrhosis, n (%) | 137 (11.5) |
| Jaundice, n (%) | 131 (11.0) |
| Body mass index, kg/m2, median (IQR) | 24.9 (22.2-27.8) |
| ALBI score, median (IQR) | -2.58 (-2.92- -2.23) |
| CA19-9 UI/mL, median (IQR) | 55.5 (18.5-271.0) |
| ***Tumor characteristics*** |  |
| Largest tumor size, cm, median (IQR) | 6.0 (4.0-8.6) |
| Number of lesions, median (IQR) |  |
| Single | 993 (83.2) |
| Multiple | 200 (16.8) |
| Tumor burden score, median (IQR) | 6.1 (4.1-8.8) |
| Morphology, periductal infiltrating type, n (%) | 168 (14.1) |
| Histological grade, poor differentiated, n (%) | 220 (18.4) |
| Lymphovascular invasion, n (%) | 414 (34.7) |
| Perineural invasion, n (%) | 276 (23.1) |
| ***Treatment data*** |  |
| Neoadjuvant therapy, n (%) | 107 (9.0) |
| Major resection, n (%) | 310 (26.0) |
| Extended hepatectomy, n (%) | 134 (11.2) |
| Vascular resection, n (%) | 244 (20.5) |
| Bile duct resection, n (%) | 160 (13.4) |
| ***Outcomes*** |  |
| Operation time, minutes, median (IQR) | 233.0 (138.0-361.0) |
| Estimated blood loss, ml, median (IQR) | 450.0 (200.0-800.0) |
| Blood transfusion, n (%) | 602 (50.5) |
| Lymphadenectomy, n (%) | 626 (52.5) |
| Lymph node metastases, n (%) | 273 (22.9) |
| Textbook Oncological Outcome, n (%) | 800 (67.1) |
| Positive resection margin, n (%) | 210 (17.6) |
| 30-day readmission, n (%) | 91 (7.6) |
| Severe complications, n (%) | 167 (14.0) |
| 90-day mortality, n (%) | 38 (3.2) |
| Length of stay, days, median (IQR) | 13.0 (8.0-18.0) |

ASA-PS: American Society of Anesthiologist Performance Status, ALBI score: albumin-bilirubin score,IQR: interqurtile range

**Supplementary Table 2.** Comparison of benchmark values between major and minor liver resections.

|  | Benchmark values | | |
| --- | --- | --- | --- |
| Parameter | Major Resection | Minor Resection | Entire Cohort |
| Number of lymph nodes retrieved | ≥2 | ≥2 | ≥3.0 |
| Estimated intraoperative blood loss, mL | ≤1000 | ≤500 | ≤600.0 |
| Perioperative blood transfusion, % | ≤71.4 | ≤45.1 | ≤42.9 |
| Operative time, min | ≤380 | ≤290 | ≤339.0 |
| Textbook Oncological Outcome, % | ≥62.1 | ≥58.3 | ≥59.3 |
| Positive margin resection, % | ≤31.6 | ≤27.5 | ≤27.5 |
| 30-day readmission, % | ≤0 | ≤5.0 | ≤3.6 |
| Severe complication, % | ≤12.5 | ≤16.7 | ≤14.3 |
| 90-day mortality, % | ≤0 | ≤1.1 | ≤4.8 |
| Postoperative hospital stay, median, days | ≤17 | ≤14 | ≤14.0 |

**Supplementary Table 3.** Comparison between patients with negative and positive resection margins among benchmark cases

|  | Negative margin | Positive margin | p-value |
| --- | --- | --- | --- |
|  | n=523 | n=77 |  |
| ***Patient demographic*** |  |  |  |
| Age, y, median (IQR) | 57.0 [48.6, 65.0] | 65.0 [56.7, 71.0] | **<0.001** |
| Sex, male, n (%) | 317 (60.6) | 30 (39.0) | **<0.01** |
| Year of surgery, 2011-2020, n (%) | 274 (52.4) | 47 (61.0) | 0.19 |
| Cirrhosis, n (%) | 76 (14.5) | 8 (10.4) | 0.42 |
| Body mass index, kg/m2, median (IQR) | 24.6 [22.0, 27.2] | 24.2 [21.8, 27.2] | 0.73 |
| ALBI score, median (IQR) | -2.73 [-2.99, -2.46] | -2.67 [-3.12, -2.25] | 0.53 |
| CA19-9, U/mL, median (IQR) | 40.9 [15.5, 182.0] | 69.7 [23.0, 225.0] | 0.07 |
| ***Tumor characteristics*** |  |  |  |
| Largest tumor size, cm, median (IQR) | 5.8 [3.9, 8.0] | 7.0 [4.5, 9.0] | 0.06 |
| Number of tumors, median (IQR) | 1 [1, 1] | 1 [1, 1] | **0.04** |
| Single | 456 (87.2) | 63 (81.8) | 0.267 |
| Multiple | 67 (12.8) | 14 (18.2) |  |
| Tumor burden score, median (IQR) | 6.1 [4.1, 8.1] | 7.1 [4.6, 9.1] | **0.03** |
| Morphology, periductal infiltrating type, n (%) | 19 (3.6) | 6 (7.8) | 0.16 |
| Histological grade, poor differentiated, n (%) | 62 (11.9) | 18 (23.4) | **0.01** |
| Lymphovascular invasion, n (%) | 130 (24.9) | 46 (59.7) | **<0.001** |
| Perineural invasion, n (%) | 58 (11.1) | 29 (37.7) | **<0.001** |

ALBI score: albumin-bilirubin score,IQR: interqurtile range
